# The impact of underreported infections on vaccine effectiveness estimates derived from retrospective cohort studies

**DOI:** 10.1093/ije/dyae077

**Published:** 2024-06-06

**Authors:** Chiara Sacco, Mattia Manica, Valentina Marziano, Massimo Fabiani, Alberto Mateo-Urdiales, Giorgio Guzzetta, Stefano Merler, Patrizio Pezzotti

**Affiliations:** ECDC Fellowship Programme, Field Epidemiology Path (EPIET), European Centre for Disease Prevention and Control (ECDC), Stockholm, Sweden; Department of Infectious Diseases, Istituto Superiore di Sanità, Rome, Italy; Center for Health Emergencies, Fondazione Bruno Kessler, Trento, Italy; Center for Health Emergencies, Fondazione Bruno Kessler, Trento, Italy; Department of Infectious Diseases, Istituto Superiore di Sanità, Rome, Italy; Department of Infectious Diseases, Istituto Superiore di Sanità, Rome, Italy; Center for Health Emergencies, Fondazione Bruno Kessler, Trento, Italy; Center for Health Emergencies, Fondazione Bruno Kessler, Trento, Italy; Department of Infectious Diseases, Istituto Superiore di Sanità, Rome, Italy

**Keywords:** Vaccine effectiveness, bias, retrospective cohort study, underreporting

## Abstract

**Background:**

Surveillance data and vaccination registries are widely used to provide real-time vaccine effectiveness (VE) estimates, which can be biased due to underreported (i.e. under-ascertained and under-notified) infections. Here, we investigate how the magnitude and direction of this source of bias in retrospective cohort studies vary under different circumstances, including different levels of underreporting, heterogeneities in underreporting across vaccinated and unvaccinated, and different levels of pathogen circulation.

**Methods:**

We developed a stochastic individual-based model simulating the transmission dynamics of a respiratory virus and a large-scale vaccination campaign. Considering a baseline scenario with 22.5% yearly attack rate and 30% reporting ratio, we explored fourteen alternative scenarios, each modifying one or more baseline assumptions. Using synthetic individual-level surveillance data and vaccination registries produced by the model, we estimated the VE against documented infection taking as reference either unvaccinated or recently vaccinated individuals (within 14 days post-administration). Bias was quantified by comparing estimates to the known VE assumed in the model.

**Results:**

VE estimates were accurate when assuming homogeneous reporting ratios, even at low levels (10%), and moderate attack rates (<50%). A substantial downward bias in the estimation arose with homogeneous reporting and attack rates exceeding 50%. Mild heterogeneities in reporting ratios between vaccinated and unvaccinated strongly biased VE estimates, downward if cases in vaccinated were more likely to be reported and upward otherwise, particularly when taking as reference unvaccinated individuals.

**Conclusions:**

In observational studies, high attack rates or differences in underreporting between vaccinated and unvaccinated may result in biased VE estimates. This study underscores the critical importance of monitoring data quality and understanding biases in observational studies, to more adequately inform public health decisions.

Key Messages

- We assess the impact of the underreported infections (i.e. under-notified and under-ascertained) on real-world vaccine effectiveness estimates in retrospective cohort studies based on surveillance data and population-based vaccination registries.
- We identified two factors that may result in pronouncedly biased vaccine effectiveness estimates, namely heterogeneities in reporting ratio between vaccinated and unvaccinated groups and high yearly infection attack rates (>50%).
- To better inform public health decisions, this study highlights the critical need of monitoring data quality and fostering research efforts aimed at a deeper understanding of the impact of different sources of bias in observational vaccine effectiveness studies.

## Introduction

Vaccination is an invaluable resource in preventing and managing infectious diseases with vaccines rigorously tested and evaluated for both efficacy and safety through well-defined experimental procedures, and ongoing monitoring of vaccine safety through surveillance systems, even after approval.

Randomized clinical trials (RCTs) are the gold standard for evaluating vaccine efficacy and safety, systematically assessing potential benefits against known or potential risks. RCTs, designed to control confounding factors and characterized by limited sample size and follow-up durations, may not fully capture real-world complexities.^1^ Therefore, vaccine effectiveness (VE) is monitored through observational studies to assess: potential differences in VE in geographical areas or subpopulations not included or underrepresented in clinical trials; changes in VE due to programmatic issues (i.e. sub-optimal cold-chain, altered intervals between doses, incomplete vaccine schedules), to pathogen evolution in the population, or under different levels of pre-existing natural immunity in the studied population; long-term changes in VE due, e.g. to the waning of immunity in individuals. Despite observational studies of real-world VE are prone to biases, they are crucial to evaluate post-implementation performance of vaccines and to timely address public health issues in terms of immunization policies and strategies.^2–4^

One of the main approaches adopted for VE observational studies is the cohort design, which exploits surveillance data, usually consisting of registries of documented infections, and population-based vaccinations registries. VE studies against laboratory-confirmed infection (or other clinical endpoints), linking individual data on confirmed cases and vaccination registries, have been performed for different diseases (e.g. COVID-19, influenza, or measles) in several countries.^5–12^ However, VE estimates from such data may present several potential sources of bias that need to be considered.^13–16^ One of these sources relates to underreported infections, due to under-notification (i.e. failure to report cases that have sought medical attention and received a diagnosis) or under-ascertainment (e.g. missed cases due to health-seeking behaviours and diagnostic capability).^17^ Infections undetected by the surveillance system may obscure the true size of the epidemic spread, leading to an underestimation of the true incidence of the infection in vaccinated and unvaccinated, ultimately biasing VE estimates obtained through cohort studies.^18–21^

Being able to assess the direction and magnitude of biases of VE estimates in the presence of underreported infections is of key importance both to advance the knowledge on their limits and potential, as well as to improve our preparedness for future pandemic challenges.

In this study, we developed an epidemic model, mimicking the spread of a vaccine-preventable respiratory disease in an initially naive population, and a vaccination campaign with a vaccine of known VE. The model generated synthetic individual-level surveillance data and vaccination registries for cohort observational studies, used to estimate VE. We established a baseline scenario with 22.5% yearly cumulative infection rate and 30% reporting ratio, equal in vaccinated and unvaccinated individuals. We assessed the impact of underreporting on VE estimates in fourteen alternative scenarios, each modifying one or more baseline assumptions.

## Methods

### The model

We developed a stochastic individual-based model (IBM) to simulate the transmission dynamics of a generic novel respiratory virus spreading in a fully naïve population (1 million individuals) and the concurrent implementation of a massive vaccination campaign. The simulated epidemic was characterized by multiple epidemic waves, mimicking the effect of seasonality or non-pharmaceutical interventions that have been observed in previous pandemics.^22–24^ Simulations are initialized with 100 infected cases and ran over a timeframe of one year. The vaccination program started at Day 50 at a constant daily rate, reaching a coverage of 80% at the end of the simulation (Day 365). We assumed a one-dose vaccine administered to non-infectious individuals only. Vaccination is assumed to provide a partial protection against infection (leaky vaccine) that wanes over time. Vaccinated individuals could be infected (breakthrough infection) with a lower relative risk compared with unvaccinated individuals and we assumed no reduction in infectiousness among breakthrough infections (Supplementary Figure S2, available as Supplementary data at *IJE* online).

To assess the potential bias of VE estimates obtained through observational studies in the presence of underreporting, we considered a baseline scenario, where the simulated epidemic resulted in a cumulative infection attack rate of about 22.5% distributed across three epidemic waves characterized by infection attack rates of approximately 15%, 3.5% and 4%, respectively (Supplementary Figure S3, available as Supplementary data at *IJE* online). At the baseline, the reporting ratio (*r*), defined as the proportion of reported infections over the total number of infections, was set at 30% and it was assumed to be the same in vaccinated and unvaccinated individuals. Each infection was labelled as ‘reported’ or not by sampling from a Bernoulli distribution with probability equal to reporting ratio. We assumed that the vaccine is not protective in the first 14 days after administration (i.e. vaccine efficacy is 0). The maximum vaccine efficacy was assumed to be achieved 14 days after administration, after which it wanes over time according to an exponential decay (see Supplementary Figure S2, available as Supplementary data at *IJE* online). Natural infection provided complete and permanent protection against re-infection. Parameter values for the baseline scenario are reported in Supplementary Table S1, available as Supplementary data at *IJE* online.

We then considered 14 alternative scenarios, in which we modified one or two of the baseline assumptions at a time, to determine in which scenario the underreporting can bias VE estimates (Table 1). Scenarios 1–2 focused on different levels of reporting, scenarios 3–4 explored heterogeneities in reporting ratio by vaccination status, whereas scenarios 5–6 investigated the impact of an epidemic characterized by higher attack rates. Finally, in scenarios 7–14, we investigated a faster waning of vaccine-induced immunity, waning of natural immunity, the combination of different assumptions on reporting and higher attack rate, and age-specific reporting ratios. All scenarios are described fully in the Supplementary Material, available as Supplementary data at *IJE* online.

**Table 1:** Simulated alternative scenarios

Differences with respect to baseline	Name of scenario	Reporting ratio (*r*)			Cumulative infection attack rate	Waning rate of vaccine immunity (*w*)	Waning of natural protection (*z*)
		Unvaccinated	Vaccinated	Age class			
–	Baseline	30%	30%	–	∼22.5%	0.005 days^−1^	None
Levels of reporting	Scenario 1	**10%**	**10%**	–	∼22.5%	0.005 days^−1^	None
	Scenario 2	**50%**	**50%**	–	∼22.5%	0.005 days^−1^	None
Heterogeneities in reporting between vaccinated and unvaccinated	Scenario 3	**25%**	**30%**	–	∼22.5%	0.005 days^−1^	None
	Scenario 4	**35%**	**30%**	–	∼22.5%	0.005 days^−1^	None
Higher infection attack rates	Scenario 5	30%	30%	–	**∼50%**	0.005 days^−1^	None
	Scenario 6	30%	30%	–	**∼75%**	0.005 days^−1^	None
Faster waning of vaccine immunity	Scenario 7	30%	30%	–	∼22.5%	**0.075 days^−1^**	None
Waning of natural protection	Scenario 8	30%	30%	–	∼22.5%	0.005 days^−1^	**0.0025 days^−1^ starting 90 days from infection**
	Scenario 9	30%	30%	–	∼22.5%	0.005 days^−1^	**0.0038 days^−1^ starting 90 days from infection**
Levels of reporting and higher infection attack rate	Scenario 10	**10%**	**10%**	–	**∼50%**	0.005 days^−1^	None
	Scenario 11	**50%**	**50%**	–	**∼50%**	0.005 days^−1^	None
Heterogeneities in reporting between vaccinated and unvaccinated and higher infection attack rate	Scenario 12	**25%**	**30%**	–	**∼50%**	0.005 days^−1^	None
	Scenario 13	**35%**	**30%**	–	**∼50%**	0.005 days^−1^	None
Heterogeneities in reporting by age	Scenario 14	**–**	**–**	**40% for ≥65 years;** **27% for <65 years**	∼22.5%	0.005 days^−1^	None

Key assumptions that are modified in each scenario with respect to the baseline are highlighted in bold.

For each scenario, we simulated 100 stochastic replicates, recording a synthetic vaccination registry and line-lists of all infections and only reported ones.

### Statistical analysis

We applied retrospective, population-based, cohort studies to the synthetic surveillance data produced by the model. The start date was set at calendar Day 120 of simulations, i.e. 70 days after the beginning of the vaccination campaign. Participants were followed up until the date of infection or the end of the follow-up period on the calendar Day 365, whichever occurred first. All the individuals with a previous reported infection before the start of the study were excluded from the analysis.

We measured the incidence of reported infections as the outcome. VE was computed for each simulation replicate from the incidence rate ratios (IRRs) of reported infections in vaccinated persons and in a reference group, as (1−IRR)×100. Point estimates and confidence intervals are given by the mean and 2.5 and 97.5 percentiles over estimates obtained from 100 stochastic replicates.

To define the reference group, we used two different approaches. The first considers ‘unvaccinated’ as reference group, including those who were not vaccinated or received the vaccine dose in the previous 2 weeks. The underlying assumption is that the infection risk in the first 14 days post-vaccination equals that of unvaccinated individuals.^25^ The reference category in the second approach consists only of those receiving the vaccine dose in the previous 2 weeks (‘recently vaccinated’).^26–28^

For each scenario, we computed two main measures of VE, which we will refer to as VE_t_ and VE_p_. VE_t_ measures the individuals’ level of protection against infection and its waning over time after vaccine administration (starting from Day 15, when the vaccine efficacy is assumed to reach its maximum), whereas VE_p_ measures the level of protection conferred by the vaccine to the entire vaccinated population in a specific period of the epidemic. If individuals in a population were all vaccinated at the same time, or if there was no waning of immunity, the two measures would coincide; in realistic cases, VE_p_ takes into account the heterogeneity in residual vaccine protection across individuals vaccinated at different times of the vaccination campaign to provide a summary measure of the population protection.

VE_t_ was estimated at 2-week intervals from vaccine administration (15–28 to 239–252 days), splitting individual time of exposure into weekly time intervals and according to vaccination status. VE_p_ was estimated at fixed time intervals of 4 weeks from the study start date (from 120–147 to 344–364 days), splitting individual time of exposure into 4-week time intervals and according to vaccination status.

To estimate the IRRs of notified infections after vaccination compared with the reference group, we applied a Poisson regression model including the vaccination status (with respect to the adopted reference group: ‘unvaccinated’ or ‘recently vaccinated’) as independent variable and the time of exposure (measured in days) as offset. To estimate VE_t_, we adjusted for the calendar week.

To investigate the magnitude and the direction of bias due to underreporting, we compared VE_t_ and VE_p_ with their reference values. The reference for VE_t_ (denoted as VE_t_*) was computed as the mean of the daily vaccine-induced protection assumed in the model (shown in Supplementary Figure S2, available as Supplementary data at *IJE* online and defined by parameters in Supplementary Table S1, available as Supplementary data at *IJE* online) considering 2-week intervals since vaccination. The reference for the period vaccine effectiveness VE_p_ (denoted by VE_p_*) could not be defined analytically and, thus, we compared VE_p_ to the period vaccine effectiveness that would be estimated assuming that all the infections were reported to the surveillance system, i.e. reporting ratio equal to 100%.

## Results

**Figure 1.**
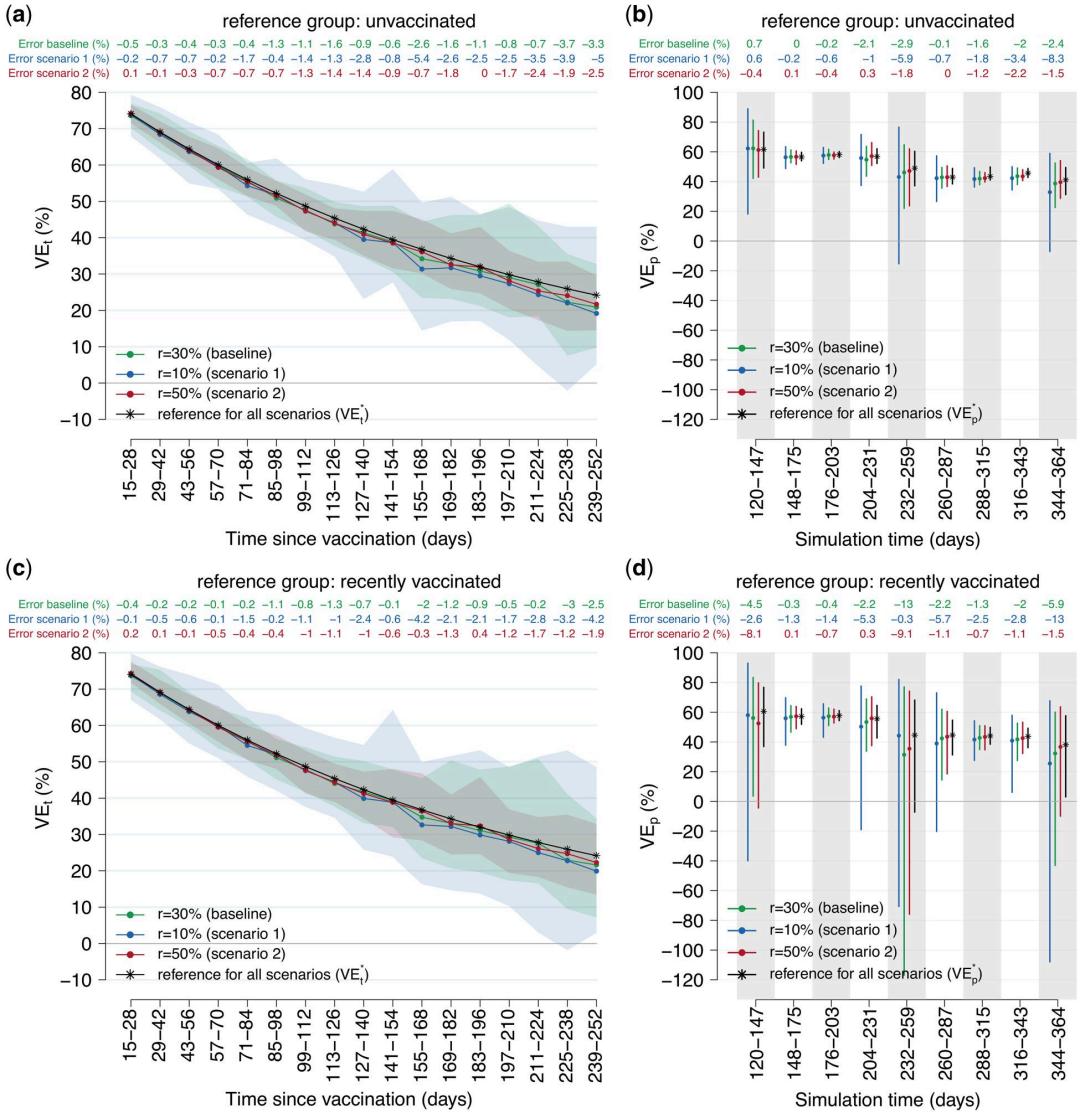
Changes in vaccine effectiveness (VE) estimates against notified infection considering lower or higher levels of reporting with respect to the baseline (scenarios 1 and 2). (a) Estimates of VE at different time intervals from vaccine administration (VE_t_) using the ‘unvaccinated’ reference group. Solid lines: mean from 100 simulations; shaded areas: 95% CI from 100 simulations. (b) Estimates of VE at different time intervals from the study start date (VE_p_) using the ‘unvaccinated’ reference group. Points: mean from 100 simulations; error bars: 95% CI from 100 simulations. (c) as (a) but using the ‘recently vaccinated’ reference group. (d) as (b) but using the ‘recently vaccinated’ reference group. ${\mathrm{VE}}_{t}^{*}$: simulated vaccine-induced protection at 2-week time intervals since vaccine administration. ${\mathrm{VE}}_{p}^{*}$: period vaccine effectiveness estimated under the assumption of reporting ratio equal to 100%

In all figures, values obtained for VE_t_* and VE_p_* will be shown in black. VE_t_* decreases over time due to the assumed waning of vaccine protection. VE_p_* also declines over time due to the population effect of individual waning immunity: since vaccination is administered at a constant daily rate, as the study progresses a higher proportion of vaccinated individuals will consist of individuals who received the vaccine earlier, resulting in a lower population protection compared with the study start. In the baseline scenario, characterized by a yearly attack rate of 22.5% and a reporting ratio equal to 30%, we found that the obtained estimates for VE_t_ and VE_p_ (green in Figure 1) were close to their reference values. The absolute error in VE_t_ estimates remained below 5% when using either the unvaccinated or the recently vaccinated as a reference group (Figure 1a and c). As for VE_p,_ the differences from the reference ranged from 0% to 3%, and from 0% to 13%, using respectively the unvaccinated and the recently vaccinated as reference groups (Figure 1b and d). The estimates of VE_p_ were characterized by high variability when the number of cases was very low, at the beginning and at the end of the study period and between epidemic waves (Supplementary Figure S3, available as Supplementary data at *IJE* online). In general, the uncertainty around the punctual estimates of both VE measures was higher when considering the recently vaccinated as reference group compared with the unvaccinated, especially for estimates of VE_p_.

Lower or higher reporting ratios (10% and 50%, respectively, scenario 1 and 2) had a negligible impact on both VE estimates (Figure 1). However, the width of the confidence interval was higher when reporting ratios were lower due to lower case counts in the analysis.

**Figure 2.**
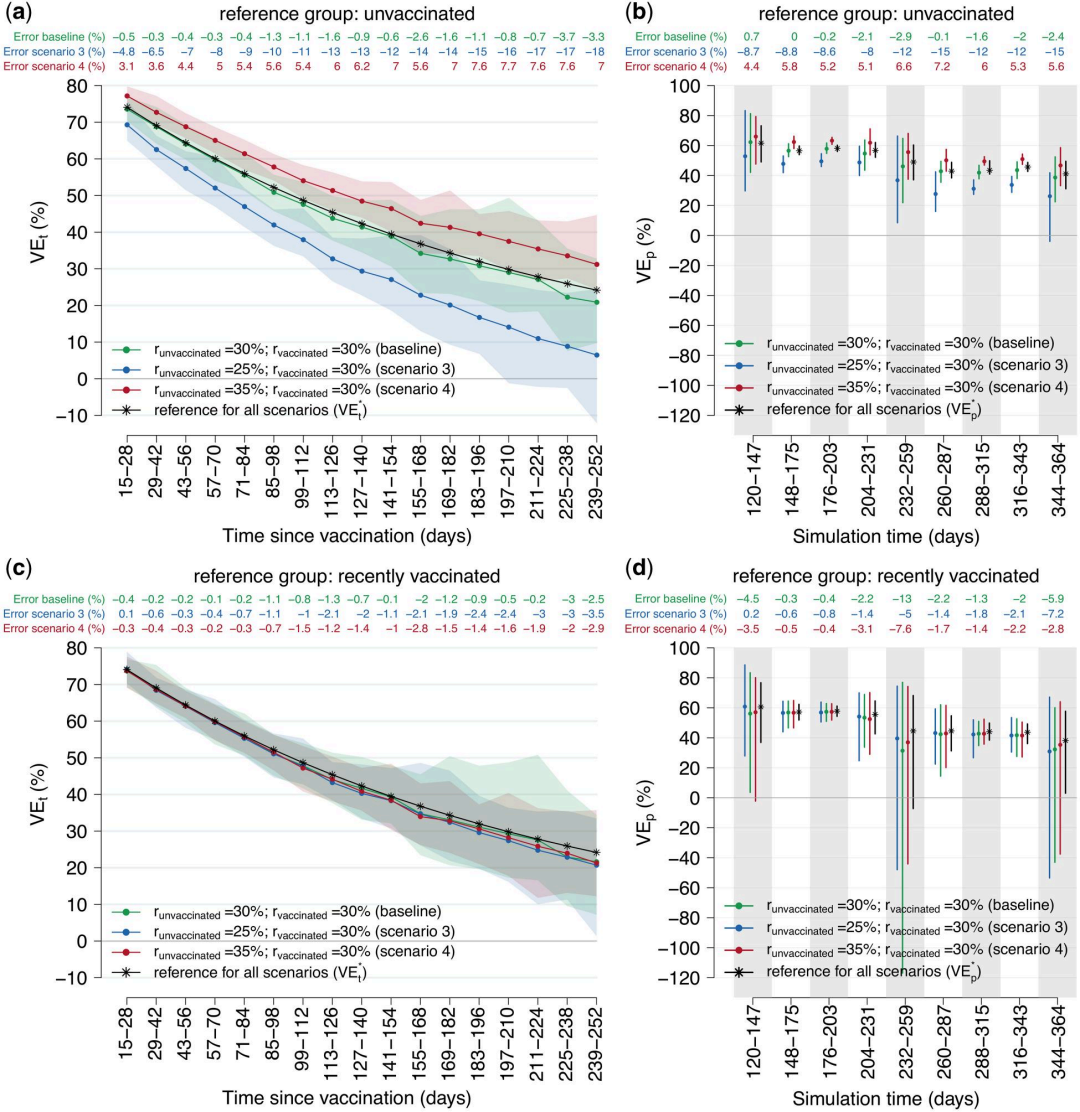
Changes in vaccine effectiveness (VE) estimates against notified infection considering heterogeneous reporting among unvaccinated and vaccinated (scenarios 3 and 4). (a) Estimates of VE at different time intervals from the vaccine administration (VE_t_) using the ‘unvaccinated’ reference group. Solid lines: mean from 100 simulations; shaded areas: 95% CI from 100 simulations. (b) Estimates of VE at different time intervals from the study start date (VE_p_) using the ‘unvaccinated’ reference group. Points: mean from 100 simulations; error bars: 95% CI from 100 simulations. (c) as (a) but using the ‘recently vaccinated’ reference group. (d) as (b) but using the ‘recently vaccinated’ reference group. ${\mathrm{VE}}_{t}^{*}$: simulated vaccine-induced protection at 2-week time intervals since vaccine administration. ${\mathrm{VE}}_{p}^{*}$: period vaccine effectiveness estimated under the assumption of reporting ratio equal to 100%

Considering heterogeneous reporting (scenario 3 and 4), using the unvaccinated as reference produced a pronounced bias in both VE_t_ and VE_p_ (Figure 2). In particular, assuming a lower reporting ratio (25%, scenario 3) in unvaccinated individuals with respect to vaccinated ones resulted in a marked underestimation of VE_t_*. Specifically, the absolute error ranged from 5% (at 15–28 days since the vaccination) to 18% (at 239–252 days since the vaccination) (Figure 2a), compared with 0–4% when using the recently vaccinated reference group (Figure 2c). When considering the unvaccinated reference group, VE_p_* was also underestimated with absolute errors comprised between 9% and 15% (Figure 2b), against 0–7% with the recently vaccinated reference group (Figure 2d). Conversely, a higher reporting ratio in unvaccinated (35%) than in vaccinated (30%) (scenario 4) resulted in an overestimation of VE_t_* and VE_p_*. In both scenarios, the difference between the estimated VE and the corresponding reference increased notably in later intervals.

As expected, in the presence of heterogeneous reporting in vaccinated and unvaccinated groups, using the recently vaccinated as reference group, instead of unvaccinated, yielded negligible bias comparable to what was observed at baseline (Figure 2c and d).

**Figure 3.**
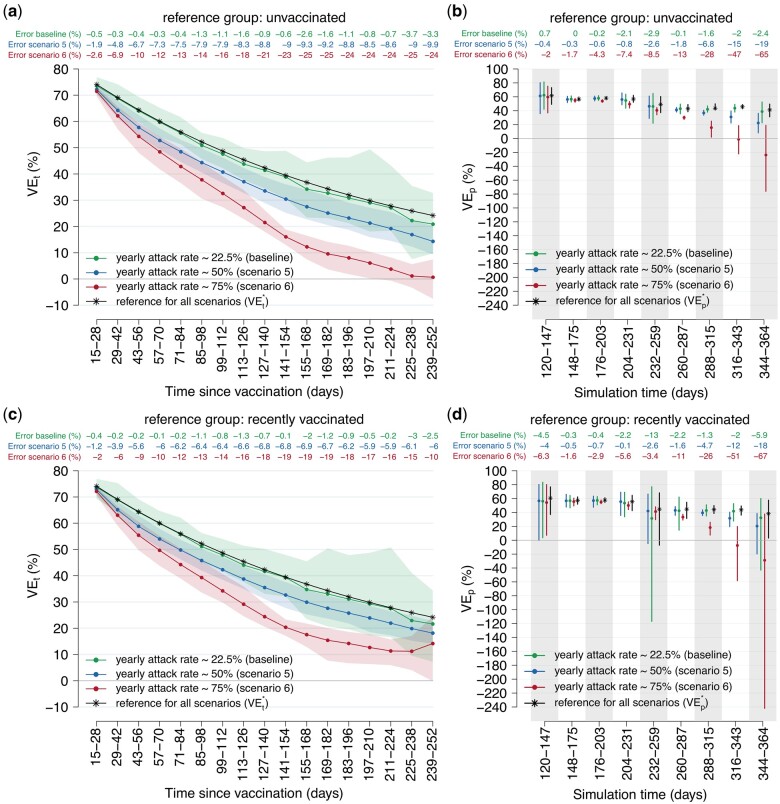
Changes in vaccine effectiveness (VE) estimates against notified infection considering higher attack rates compared with the baseline (scenarios 5 and 6). (a) Estimates of VE at different time intervals from the vaccine administration (VE_t_) using the ‘unvaccinated’ reference group. Solid lines: mean from 100 simulations; shaded areas: 95% CI from 100 simulations. (b) Estimates of VE at different time intervals from the study start date (VE_p_) using the ‘unvaccinated’ reference group. Points: mean from 100 simulations; error bars: 95% CI from 100 simulations. (c) as (a) but using the ‘recently vaccinated’ reference group. (d) as (b) but using the ‘recently vaccinated’ reference group. ${\mathrm{VE}}_{t}^{*}$: simulated vaccine-induced protection at 2-week time intervals since vaccine administration. ${\mathrm{VE}}_{p}^{*}$: period vaccine effectiveness estimated under the assumption of reporting ratio equal to 100%

Results from the alternative attack rate scenarios, suggest that if the reporting is homogeneous in vaccinated and unvaccinated groups, extremely high attack rates may result in downward biases in VE estimates (Figure 3). Specifically, with a yearly attack rate around 50% (scenario 5), we observed an underestimation of both measures of VE: the absolute error for VE_t_ ranged from 2% (at 15–28 days since the vaccination) to 10% (at 239–252 days since the vaccination) (Figure 3a), whereas the absolute error for VE_p_ ranged from 0% to 19% (Figure 3b). As in the previous scenarios, higher bias occurred at latest time intervals and the analysis taking as reference group the ‘recently vaccinated’ was characterized by higher variability. The underestimation of VE estimates was even higher when considering the extreme case of a yearly attack rate of 75% (scenario 6).

Considering a faster waning of vaccine-induced immunity (scenario 7), the waning of natural immunity (scenario 8–9), or *r* varying by age resulted in a slight bias in VE estimation (scenario 14) (Figures S5, S6, and S9).

Assuming high attack rate, lower or higher reporting ratios (10% and 50%, respectively, scenario 10 and 11) result in a more pronounced, though still negligible, bias in VE estimation compared with scenarios 1–2 (Supplementary Figure S7, available as Supplementary data at *IJE* online). Combining assumptions that both contribute to underestimating VE (e.g. high attack rate with lower reporting in unvaccinated compared with vaccinated, scenario 12) exacerbated VE underestimation. Conversely, pairing assumptions with opposite effects (e.g. high attack rate with higher reporting in unvaccinated compared with vaccinated, scenario 13) led to a partial compensation of the two biases (Supplementary Figure S8, available as Supplementary data at *IJE* online). Further details on results of scenarios 7–14 are reported in the Supplementary Material, available as Supplementary data at *IJE* online.

## Discussion

Our findings highlight that underreporting of infections may impact both measures of VE: VE_t_ accounting for the individuals’ immunity against infection and its loss over time since vaccine administration, VE_p_ accounting for the overall level of protection against infection of the vaccinated population in different periods. In general, estimates of VE_p_ and VE_t_ followed similar patterns over time, although VE_p_ exhibited greater variability, especially at the beginning or end of the study period. In the presence of underreported infections, bias in VE_t_ and VE_p_ estimates may arise because individuals who had an undetected infection are no longer at risk of infection but will be mistakenly included in the population at risk. This affects both the vaccinated and their reference group (unvaccinated or recently vaccinated), however, since the vaccine provides protection against infection individuals with prior unreported infections are more likely to be in the reference population than in the vaccinated one.^29^ This leads to a faster decline of VE estimates compared with the reference in most scenarios considered, consistently with previous studies.^7^^,^^29–31^ Our findings confirm that underreporting exerts a cumulative impact over time on VE estimates: as the vaccination campaign progresses and the protection from natural infection builds up, a higher proportion of unvaccinated (or recently vaccinated) individuals acquires natural immunity, leading to a more marked bias in the latest time intervals.

Our results suggest that, with low attack rates and homogeneous reporting, the impact of underreporting on VE estimates is negligible. However, when considering higher levels of pathogen circulation the bias in VE estimates increases. Indeed, these scenarios are characterized by a higher number of infections, and in turn by a more marked overestimation of the population at risk at each time interval. These findings suggest that the fast waning of VE estimated in multiple retrospective observational studies for the SARS-CoV-2 Omicron variant^32^ may have been partly exaggerated by the widespread and sustained circulation of the variant.

Our findings suggest that also unbalanced reporting ratios in vaccinated and unvaccinated may considerably bias VE estimates, when taking unvaccinated as reference. Even mild differences (±5%) in reporting ratios resulted in remarkable under- or over-estimation of VE (if reporting is lower in the unvaccinated or in the vaccinated population, respectively). Heterogeneities in reporting are likely to exist and may arise from many causes, including different symptomatic rates and testing behaviours, as suggested by several studies on the COVID-19 epidemic.^33^^,^^34^ However, the direction of such differences is hard to predict: a research on COVID-19 in Australia showed that vaccinated individuals were twice as likely as those unvaccinated to report testing intentions, pointing to higher reporting ratios among vaccinated^35^; conversely, a study in Finland showed substantially lower COVID-19 testing rates among vaccinated individuals.^36^ Considering ‘recently vaccinated’ as reference group could significantly alleviate the bias originating from these heterogeneities, but may result in higher variability of VE estimates, likely due to the small sample size and short observation time of this group. This approach might also help to avoid biases stemming from unknown or unmeasured differences in risk behaviour between vaccinated and unvaccinated that could be particularly relevant at the beginning of the vaccination campaign when targeting the vulnerable population. Furthermore, this method proves particularly valuable in situations where the vaccination coverage is high, yet a new vaccine requiring VE evaluation is introduced.

Our results should be interpreted in light of the following limitations. First, the synthetic population consists of about 1 million individuals, considering smaller or larger population sizes may respectively increase or decrease the uncertainty around VE estimates. We also assumed a stable population size over the simulation period (one year), and neglected heterogeneities in transmission across ages or geographical areas. Although these are strong simplifications of disease dynamics, we aimed at providing general insights on possible bias impacting VE estimates, through a novel framework leveraging synthetic surveillance and vaccination data.

Secondly, we focused on the impact of underreporting on VE in a retrospective cohort study design. Although this design has been widely used in several countries to measure the real-word vaccine-induced protection against SARS-CoV-2^5–8^^,^^11^^,^^12^ or other respiratory viruses,^37–39^ test-negative designs based on modified case-control designs have become an increasingly popular alternative.^40^ Thirdly, although we explored scenarios with waning of the natural immunity over time, our study lacks the capability to provide insights into evaluating the impact of underreporting on the estimation of the hybrid immunity. It may be worth extending our framework to explore the impact of underreporting in alternative study designs as well as clarifying its role in measuring hybrid immunity.

Fourth, we did not consider other potential confounders possibly associated with both vaccination and infection, such as risk behaviours and comorbidities, which might introduce a further bias in VE estimates. Finally, we simulated an epidemic with a concurrent massive vaccination campaign, although the point in the epidemic trajectory when the vaccine is introduced could influence the extent and the magnitude of the bias.^29^

The constant evolution of the epidemiological situation during the COVID-19 pandemic has shown the importance of performing repeated real-world studies of VE, to monitor the vaccine-induced protection against different clinical endpoints, as well as the need for high-quality health data to obtain robust VE estimates. However, few studies focused on the evaluation of the impact of under-notification and under-ascertainment on the estimation of VE. One study considered some potential sources of bias in linked registry studies, including incomplete reporting, errors in linking individuals, and possible overestimation/underestimation of the population size of the study catchment area, showing that if multiple sources of errors occur the VE estimates at a single time point can be strongly biased.^13^

In this work, we propose a novel framework that leverages an IBM paired with statistical models to illustrate how underreporting influences VE estimates. Furthermore, in comparison to the state-of-art, this study explores estimates of VE over time, rather than focusing on solely a single time point. Additionally, the study compares the performance of two approaches based on different definitions of the reference group.

According to our findings, the direction of the bias due to underreporting under specific conditions is predictable. Our results highlight that VE estimates obtained through observational studies should be interpreted with caution, especially in the presence of signals of possible heterogeneities in reporting in vaccinated and unvaccinated individuals and high circulation of the pathogen. Post-introduction evaluation of VE is crucial to timely address public health issues. To provide more reliable indications for public health decision-makers, it is of critical importance to monitor data quality and expand the understanding on the role of different sources of bias in observational VE studies.

## Disclaimer

The author Chiara Sacco is a fellow of the ECDC Fellowship Programme, supported financially by the European Centre for Disease Prevention and Control. The views and opinions expressed herein do not state or reflect those of ECDC. ECDC is not responsible for the data and information collation and analysis and cannot be held liable for conclusions or opinions drawn.

## Ethics approval

Ethical approval is not applicable, because this article does not contain any studies with human or animal subjects.

## Supplementary Material

dyae077_Supplementary_Data

## Data Availability

Code and input data used in this study are available on Zenodo at https://doi.org/10.5281/zenodo.10782616.
