# Supplementary material for: The impact of underreported infections on vaccine effectiveness estimates derived from retrospective cohort studies
Source: Int J Epidemiol. 2024 Jun 6;53(3):dyae077. doi: 10.1093/ije/dyae077 (PMC11157963; doi:10.1093/ije/dyae077)
Supplement: dyae077_Supplementary_Data [file dyae077_supplementary_data.pdf]

Supplementary Material

The impact of underreported infections on vaccine effectiveness estimates derived from retrospective cohort studies.

Chiara Sacco[1,2] *†, Mattia Manica[3] †, Valentina Marziano[3] †, Massimo Fabiani[2], Alberto Mateo Urdiales[2], Giorgio Guzzetta[3], Stefano Merler[3] ‡, Patrizio Pezzotti[2] ‡

[1] ECDC Fellowship Programme, Field Epidemiology path (EPIET), European Centre for Disease Prevention and Control (ECDC), Stockholm, Sweden

[2] Department of Infectious Diseases, Istituto Superiore di Sanità, Rome, Italy;

[3] Center for Health Emergencies, Fondazione Bruno Kessler, Trento, Italy.

* Corresponding author. Viale Regina Elena, 299, 00161 Rome, Italy. E-mail: chiara.sacco@iss.it

†These authors contributed equally to this work.

‡ Joint senior authors

Contents

Methods **2**

The epidemic model 2

Alternative scenarios 5

Programming language and computational time 7

Additional figures and results **8**

Methods

The epidemic model

We developed a stochastic individual-based model (IBM) to simulate the transmission dynamics of a respiratory virus with pandemic potential in a fully naïve population and the concurrent implementation of a single-dose vaccination campaign.

Specifically, we considered a closed synthetic population (N) of about 1 million individuals, mixing homogenously, where each individual j in the population is characterized by the following features:

- an epidemiological status: not infectious (H) or infectious (I);
- a time T_j^I representing the time of (last) infection (if already infected);
- a time T_j^R representing the time at which the infection is reported to the surveillance system (if reported);
- a time T_j^V representing the time of vaccination (if vaccinated).

At each time t, three sets of events are simulated: new infections, new recoveries, and vaccine administration.

New infections

For each time t, we determine the number $C(t)$ of not infectious individuals (epidemiological status: H) experiencing a contact with infectious ones (epidemiological status: I) by sampling from a Poisson distribution with mean $\lambda(t)N_H(t)$, i.e.:

$$C(t) \sim Pois(\lambda(t)N_H(t))$$

where

- $N_H(t)$ represents the number of not-infectious individuals at time t;
- $\lambda(t) = \beta(t)N_I(t)/N$ is the time-dependent force of infection, where $N_I(t)$ represents the number of infectious individuals at time t, N is the total population, and $\beta(t)$ is a time-dependent transmission rate.

To simulate an epidemic trajectory characterized by multiple waves, we assumed a periodic functional form for the transmission rate (Figure S1, Table S1):

$$\beta(t) = a + b(t + c) + d \sin\left(\frac{t + e}{f}\right)$$

To determine the number of infectious contacts resulting in effective transmission of the pathogen, we randomly choose C(t) individuals from the population of non-infectious (H) and, for each selected individual j, we determine if the infectious contact will result in infection or not, by sampling from a Bernoulli distribution with probability $\chi_j(t) \in [0,1]$ representing the individual's susceptibility to infection. The relative susceptibility to infection of individual j at time t is defined as follows:

$$\chi_j(t) = 1 - \max(0, p_j^I(t), p_j^V(t))$$

where:

- $p_j^I(t)$ represents the individual's protection provided from natural infection at time t. In the baseline analysis, we assumed a full and long-lasting protection after natural infection, i.e., $p_j^I(t) = 1$ if $t \geq T_j^I$, otherwise $p_j^I(t) = 0$;
- $p_j^V(t)$ represents the individual's protection provided from vaccination at time t. We assume that the vaccine takes d days from administration to ramp-up (i.e. $p_j^V(t)$=0 if $t < T_j^V + d$) and to achieve its initial effectiveness ϑ, which wanes exponentially with a rate w:

  $p_j^V(t)=\vartheta e^{-w(t-T_j^V-d)}$ if $t \geq T_j^V + d$.

Upon effective transmission of the pathogen, the epidemiological status of the individual is updated to I (infectious) and the time of infection T_j^I is recorded.

We assume that every new infection has a probability r of being reported to the surveillance system and, if reported, the time of reporting T_j^R is also recorded, considering a Poisson distributed notification delay of mean d_r.

New recoveries

For each time t, we randomly sample the number of infectious individuals that recover from infection from a Poisson distribution of mean $\gamma N_I(t)$, where γ is the recovery rate. The epidemiological status of recovered individuals is updated from I to H (not infectious).

Vaccine administration

We implement a single-dose vaccination campaign from day 50, targeting not-infectious individuals with a constant vaccination rate ω. Vaccinated individuals are selected among not-infectious individuals that have never received a vaccine dose before (i.e. T_j^V not assigned). Upon vaccination, the individual's time of vaccination T_j^V is recorded.

Model simulation

We consider a timeframe of one year and perform simulations with a daily time step. Simulations are initialized with 100 infectious individuals. We simulated 100 stochastic runs of the individual based model and record for each simulation the line-list of infections occurring over the entire simulation timeframe and a synthetic vaccination registry.

The vaccination registry includes for each vaccinated individual, an identification code and the day of vaccination. The line-list of daily infections reports for every infection: the identification code, necessary to match the line-list with the vaccination registry, the day of infection and the day of reporting (if any).

We explored parameters' values representative of a potential epidemic characterized by a novel virus spreading in a population with no prior immunity, characterized by multiple waves, and by the implementation of a massive vaccination campaign.

Specifically, in the baseline scenario, we chose the parameters shaping the transmission rate to obtain three epidemic waves resulting in a cumulative infection attack rate over one year of about 22.5% (see Table S1, Figure S1 and S3) and assumed lifelong duration of natural immunity (Figure S2).

We modeled the implementation of a single-dose vaccination campaign with a constant daily vaccination rate of 2.5 doses per 1,000 population with a vaccine providing partial protection against infection and waning over time (Table S1 and Figure S2). Under the assumed daily vaccination rate, a coverage of about 80% is achieved by the end of the simulation. In the baseline scenario, we also consider a reporting rate of new infections equal to 30%, constant over the simulated period and across vaccinated and unvaccinated individuals. Parameter values for the baseline scenario are reported in Table S1.

Table S1. Model parameter values for the baseline scenario

	**Model parameter**	**Description**	**values**
Epidemic	a	Parameter shaping the transmission rate $\beta(t)$, see Eq. 1	0.15
	b	Parameter shaping the transmission rate $\beta(t)$, see Eq. 1	0.0005
	c	Parameter shaping the transmission rate $\beta(t)$, see Eq. 1	6
	d	Parameter shaping the transmission rate $\beta(t)$, see Eq. 1	0.16
	e	Parameter shaping the transmission rate $\beta(t)$, see Eq. 1	2
	f	Parameter shaping the transmission rate $\beta(t)$, see Eq. 1	20
	γ	Recovery rate (1/day)	0.17
Vaccination	ω	Daily vaccination rate	2.5 doses per 1,000 individuals
	ϑ	Vaccine efficacy at 15 days from inoculation	0.765
	w	Waning rate of vaccine (1/day)	0.005
Reporting	r	Reporting ratio	30%
	d_r	Reporting delay (day)	8

Alternative scenarios

We defined 14 alternative scenarios in which we varied one or more hypothesis at the time. Specifically, we considered:

- a lower reporting ratio with respect to the baseline, $r = r_{unvaccinated} = r_{vaccinated} = 10\%$ (scenario 1);
- a higher reporting ratio with respect to the baseline, $r = r_{unvaccinated} = r_{vaccinated} = 50\%$ (scenario 2);
- a lower reporting ratio of infections in unvaccinated individuals $r_{unvaccinated} = 25\%$ with respect to vaccinated $r_{vaccinated} = 30\%$ (scenario 3);
- a higher reporting ratio of infections in unvaccinated individuals $r_{unvaccinated} = 35\%$ with respect to vaccinated $r_{vaccinated} = 30\%$ (scenario 4);
- an epidemic characterized by a more marked third epidemic wave compared to the baseline, resulting in a cumulative attack rate about 50%. To this aim, from the simulation day 140, we changed the parameters a and b shaping the transmission scale factor $\beta(t)$ to $a = 0.06$ and $b = 0.001025$ (see Figure S1) (scenario 5).
- an epidemic characterized by a more marked third epidemic wave compared to the baseline, resulting in a cumulative attack rate about 75%. To this aim, from the simulation day 140, we changed the parameters a and b shaping the transmission scale factor $\beta(t)$ to $a = 0.039$ and $b = 0.0015$ (see Figure S1) (scenario 6);
- a faster waning of the protection provided by vaccine-induced immunity ($w = 0.0075$ days^{-1}) compared to the baseline, see Figure S2 (scenario 7);
- waning of protection from natural immunity starting 90 days from infection, i.e.,

  $$p_j^I(t) = \begin{cases} 0 & if\ t < T_I \\ 1 & if\ T_I \leq t \leq T_I + 90 \\ e^{-z\left(t - T_j^I - 90\right)} & if\ t > T_I + 90 \end{cases}$$

  where z= 0.0038 days^{-1} is the waning rate of the natural immunity, see Figure S2B (scenario 8).
- a slower waning of protection from natural immunity compared to scenario 8, *i.e.* $z = 0.0025$ days^{-1}, see Figure S2B (scenario 9). For this and the previous scenario, we assumed that the immunity conferred by infection is higher and declines slower over time than the protection induced by vaccination (https://doi.org/10.1016/S0140-6736(22)02465-5) and that the individual protection given from hybrid immunity (i.e. the combination of vaccination and one or more infections) at time t is equal to the maximum between the vaccine-induced and the natural immunity that the individual has at that time, taking into account the time elapsed since vaccination and the natural infection.
- a lower ($r = r_{unvaccinated} = r_{vaccinated} = 10\%$, Scenario 10) or higher ($r = r_{unvaccinated} = r_{vaccinated} = 50\%$, Scenario 11) reporting ratio with respect to baseline while assuming an epidemic characterized by a more marked third epidemic wave compared to the baseline, resulting in a cumulative attack rate about 50%.
- a lower reporting ratio of infections in unvaccinated individuals $r_{unvaccinated} = 25\%$ with respect to vaccinated $r_{vaccinated} = 30\%$ while assuming an epidemic characterized by a more marked third epidemic wave compared to the baseline, resulting in a cumulative attack rate about 50% (Scenario 12)

- a higher reporting ratio of infections in unvaccinated individuals $r_{unvaccinated}$ = 35% with respect to vaccinated $r_{vaccinated}$ = 30% while assuming an epidemic characterized by a more marked third epidemic wave compared to the baseline, resulting in a cumulative attack rate about 50% (Scenario 13)
- a higher reporting ratio of infections (40% against 27%) in elderly (>65 years old) while assuming the 2022 age distribution retrieved from EuroStat, https://doi.org/10.2908/TPS00028, (Scenario 14)

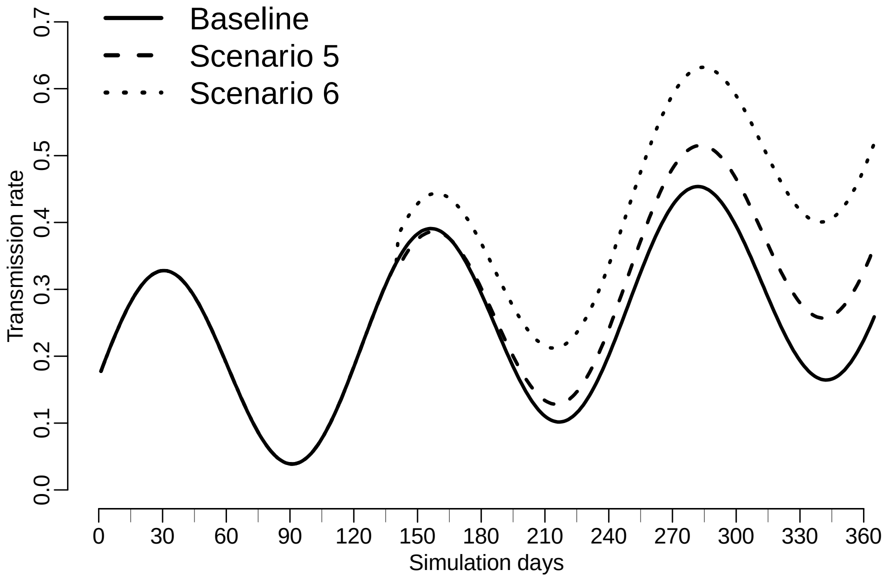

Figure S1. Transmission rate for baseline, scenario 5 and scenario 6

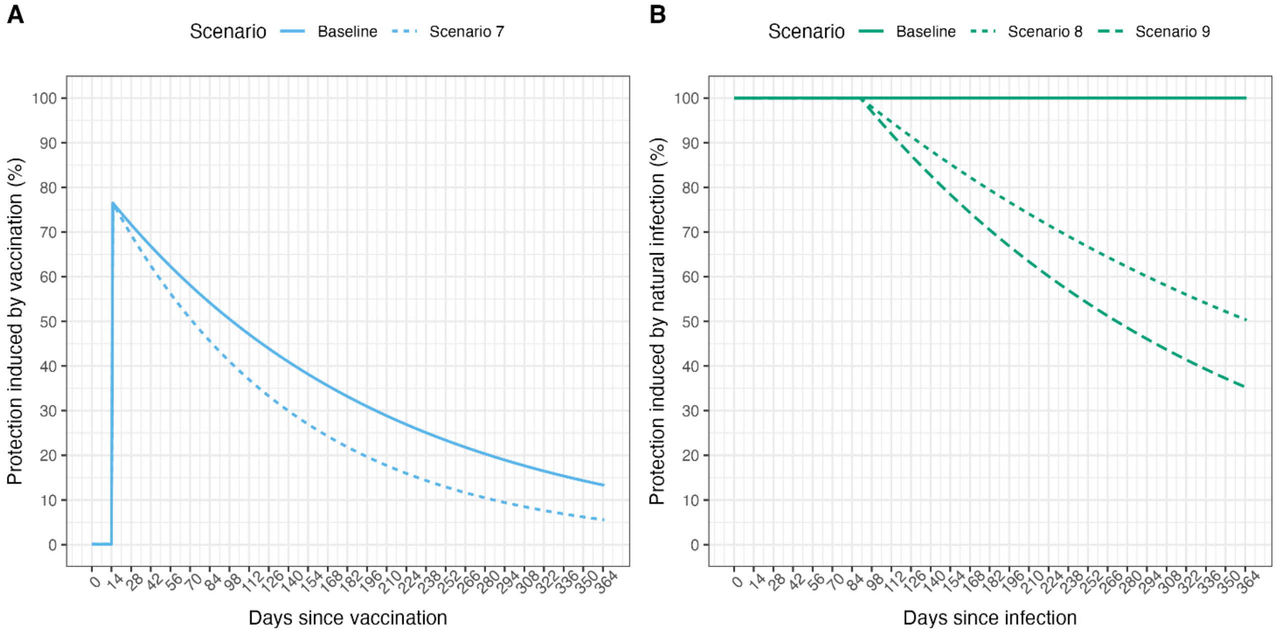

Figure S2. Waning of vaccine-induced and natural immunity. On the y-axis the protection from infection, on the x-axis the day from the event (i.e. vaccine inoculation or an infection). Left panel) waning of vaccine-induced immunity in the baseline scenario and in scenario 7. Right panel) waning of natural immunity in the baseline scenario (no waning) and in alternative scenarios 8 and 9.

Programming language and computational time

The individual-based model for simulating the epidemic was implemented in C programming language. The computational time required to run one single simulation of each scenario is about 18 seconds on a laptop with 16GB of RAM and an Apple M1 processor. The statistical data analysis pipeline was implemented in R and run on a cloud environment of the Italian National Health Institute with 256 GB of RAM and Intel(R) Xenon(R) GOld 633GR. The time to compute estimates of the vaccine effectiveness at different time intervals from the vaccine administration (VE_t) and at different time intervals from the study start date (VE_p) associated to one single simulation of each scenario was approximately 20 minutes.

Additional figures and results

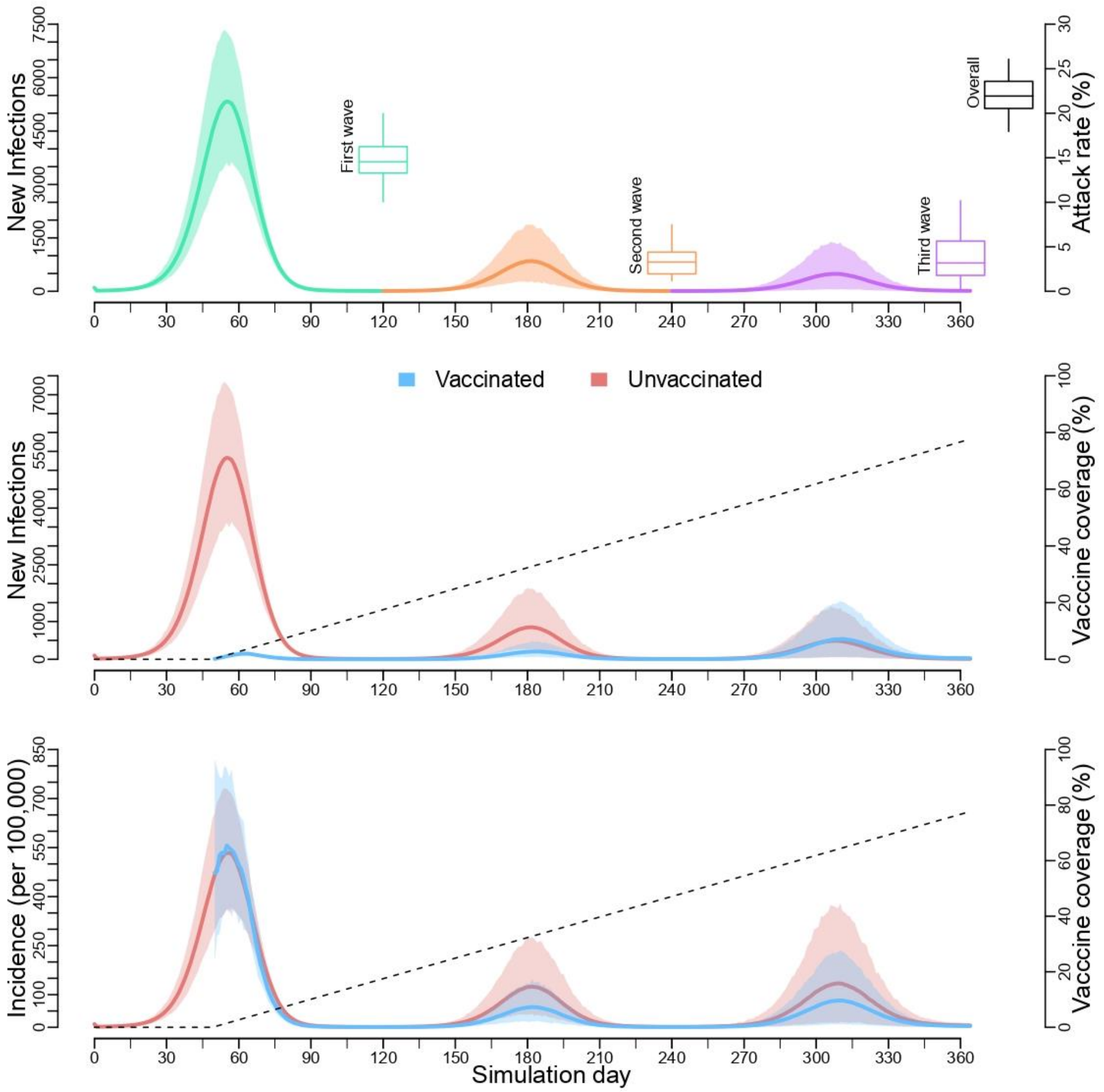

Figure S3. Epidemiological characteristics of the epidemic simulated in the baseline scenario.

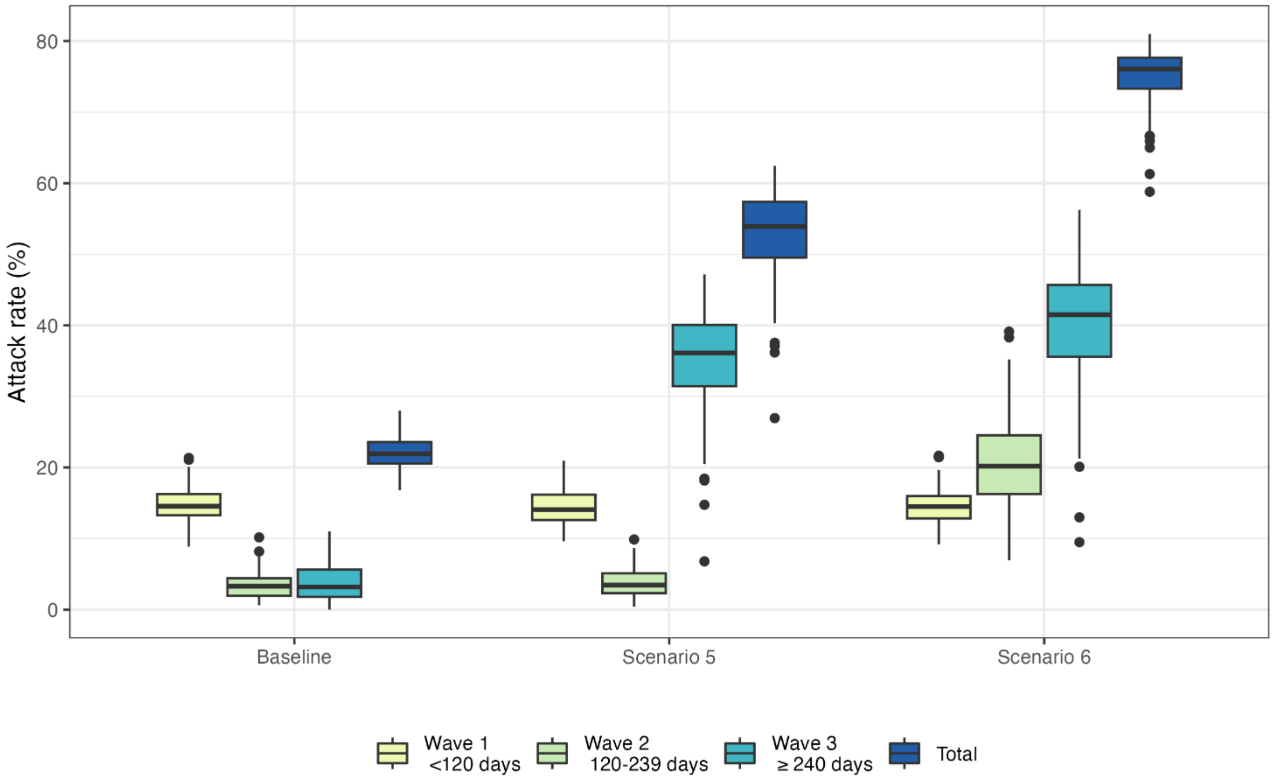

Figure S4. Boxplot of the attack rate observed in the 100 simulations of the individual based model for baseline scenario, scenario 5 and 6 during the whole simulated epidemic and in the three waves. On the y-axis the attack rate (%), on the x-axis the scenarios. Colours represent the period when the attack rate is computed.

Assuming a faster waning for vaccine protection (scenario 7) resulted in a slight underestimation of the vaccine effectiveness (VE) compared to the baseline (Figure S5). This change in the waning function seemed to affect the punctual estimates of VE_t at later calendar intervals especially when using the unvaccinated reference group. Finally, a waning of protection from naturally acquired immunity (scenarios 8 and 9) resulted in a negligible bias of VE estimates at baseline attack rates (Figure S6). VE estimates in the latter scenarios are characterized by narrower confidence intervals, possibly due to a lesser depletion of susceptibles, as we assumed that after 90 days from the infection, each individual is again in the susceptible group but with partial protection.

Considering a higher attack rate compared to the baseline and a reporting ratio equal to 50% (scenario 11), results in downward biases in both measures of VE (VE_t and VE_p) using both the unvaccinated and the recently vaccinated as reference groups (Figure S7). Specifically, the absolute error for VE_t ranged from 2% (at 15-28 days since the vaccination) to 11% (at 239-252 days since the vaccination) (Figure S7A), whereas the absolute error for VE_p ranged from 1% to 22% (Figure S7B), using the unvaccinated as reference group.

In scenario 12 and 13, where reporting ratios are heterogeneous between vaccinated and unvaccinated and the yearly attack rate is around 50%, we observed a different impact of the underreporting of the VE estimates compared scenarios 3-4 characterized by heterogeneous reporting ratios and attack rate around 22.5%.

Specifically, using the unvaccinated as reference group and assuming a yearly attack rate of 50%, with a lower reporting ratio in unvaccinated individuals with respect to vaccinated ones (scenario 12), resulted in a marked underestimation of VE_t^*. The absolute error ranged from 7% (at 15-28 days since the vaccination) to 26% (at 239-252 days since the vaccination) (Figure S8A), compared to 5%-18% at the baseline attack rate (Figure 2A). VE_p^* was also underestimated with absolute errors comprised between 10% to 33% (Figure S8B), against 9%-15% assuming a lower attack rate (Figure 2B). Conversely, the effect of a high attack rate combined with higher reporting in unvaccinated compared to vaccinated (scenario 13) appear to counterbalance each other resulting in VE_t and VE_p estimates that are only slightly biased.

Assuming a reporting ratio varying by age group (27% and 40% for those under 65 years old and for those aged 65 years and older, respectively, scenario 14) had a negligible impact on the estimates of both VE_t and VE_p (blue in Figure S9). The VE estimates for scenario 14 overlapped with those obtained in the baseline scenario (green in Figure S9).

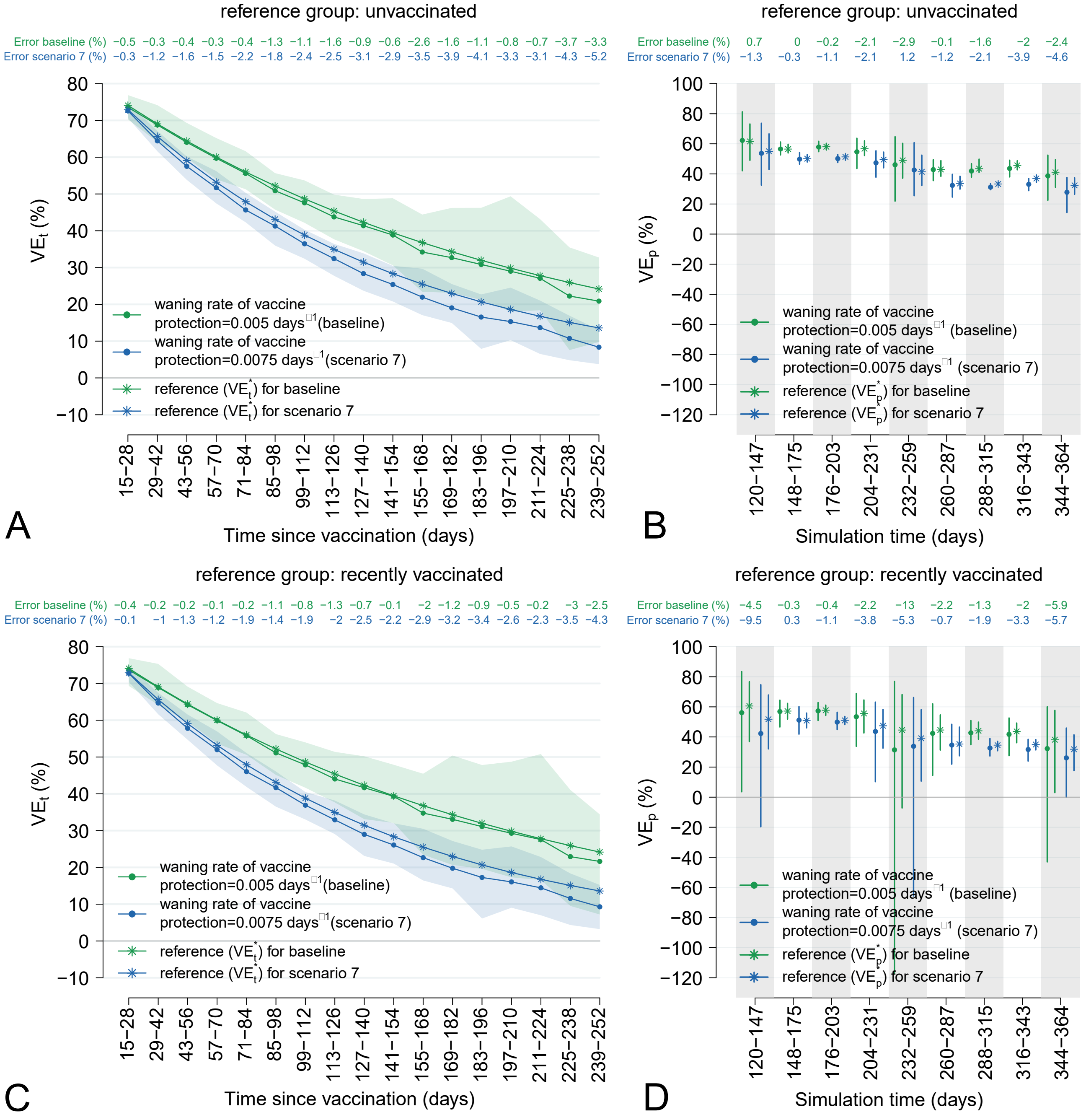

Figure S5. Changes in vaccine effectiveness (VE) estimates against notified infection considering a faster waning of vaccine protection (scenario 7). (A) Estimates of VE at different time intervals from the vaccine administration (VE_t) using the "unvaccinated" reference group. Solid lines: mean from 100 simulations; shaded areas: 95%CI from 100 simulations. Dotted lines: reference values of VE_t for the baseline and for scenario 7. (B) Estimates of VE at different time intervals from the study start date (VE_p) using the "unvaccinated" reference group. Points: mean from 100 simulations; error bars: 95%CI from 100 simulations. (C) as (A) but using the "recently vaccinated" reference group. (D) as (B) but using the "recently vaccinated" reference group. VE_t^*: simulated vaccine-induced protection at two-week time intervals since vaccine administration. VE_p^*: period vaccine effectiveness estimated under the assumption of reporting ratio equal to 100%.

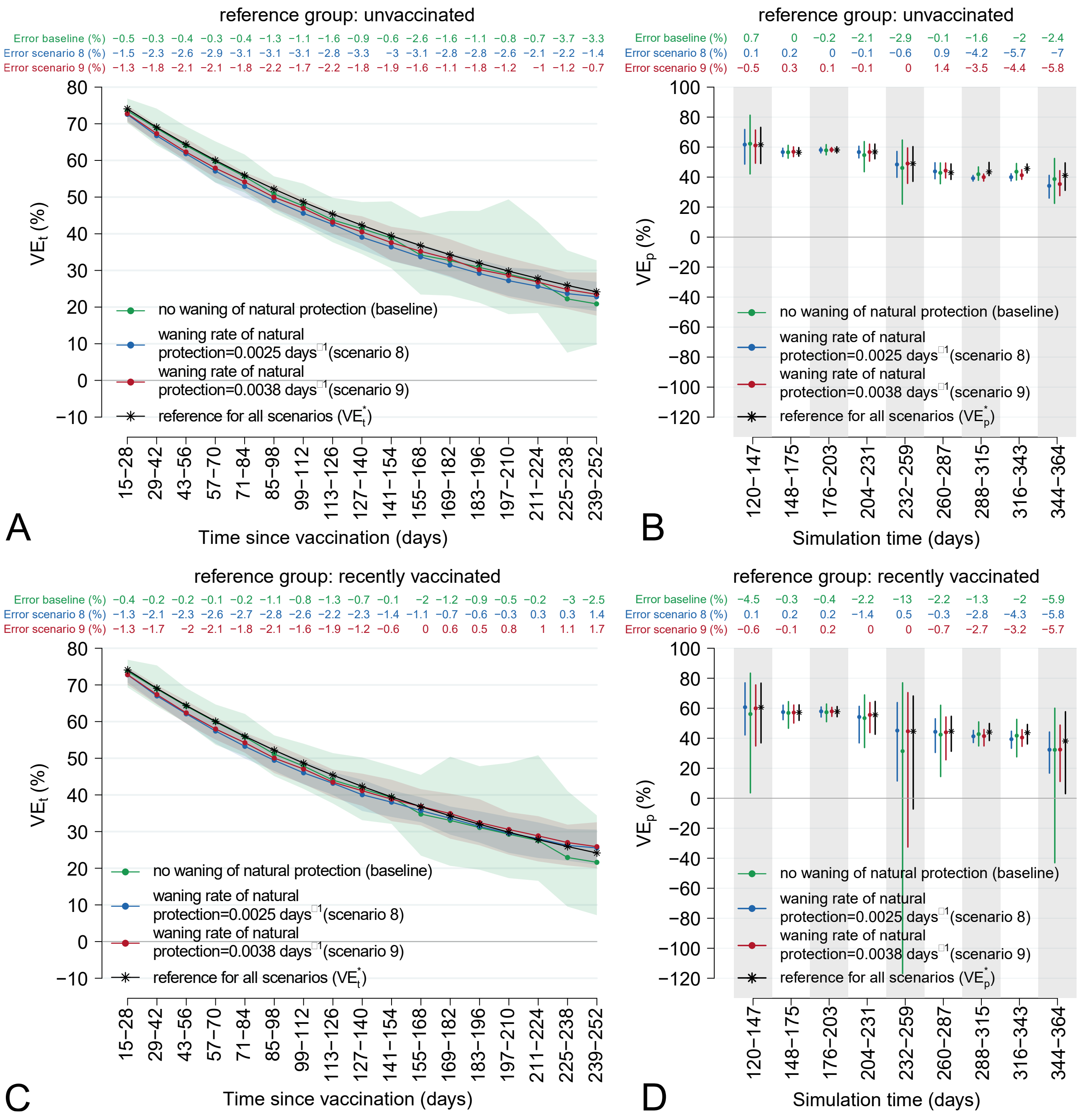

Figure S6. Changes in vaccine effectiveness (VE) estimates against notified infection considering a slow or fast waning of natural protection (scenario 8 and 9). (A) Estimates of VE at different time intervals from the vaccine administration (VE_t) using the "unvaccinated" reference group. Solid lines: mean from 100 simulations; shaded areas: 95%CI from 100 simulations. (B) Estimates of VE at different time intervals from the study start date (VE_p) using the "unvaccinated" reference group. Points: mean from 100 simulations; error bars: 95%CI from 100 simulations. (C) as (A) but using the "recently vaccinated" reference group. (D) as (B) but using the "recently vaccinated" reference group. VE_t^*: simulated vaccine-induced protection at two-week time intervals since vaccine administration. VE_p^*: period vaccine effectiveness estimated under the assumption of reporting ratio equal to 100%.

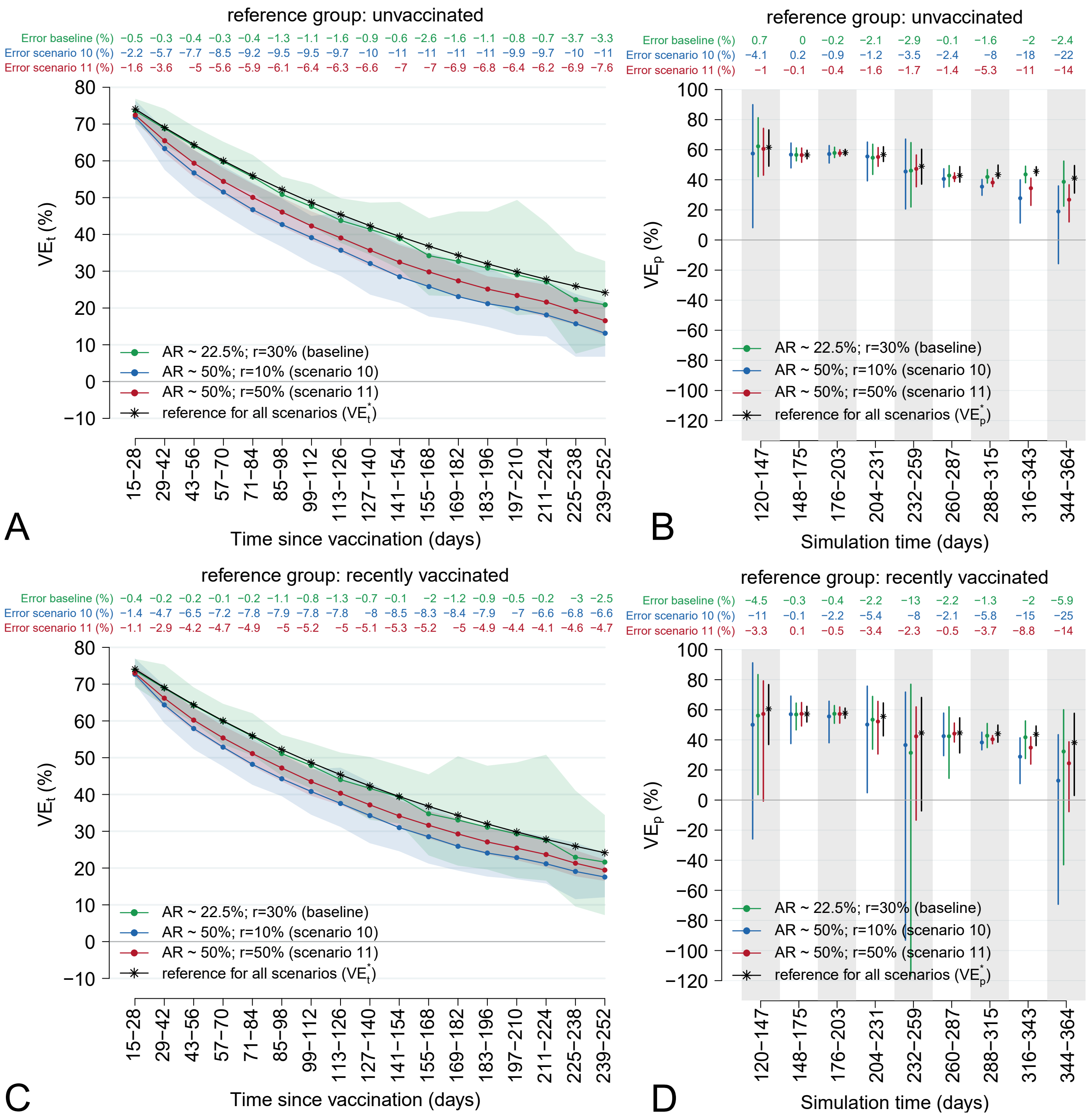

Figure S7. Changes in vaccine effectiveness (VE) estimates against notified infection considering different levels of reporting ratio and higher attack rate (50%) compared to the baseline (scenario 10 and 11). (A) Estimates of VE at different time intervals from the vaccine administration (VE_t) using the "unvaccinated" reference group. Solid lines: mean from 100 simulations; shaded areas: 95%CI from 100 simulations. (B) Estimates of VE at different time intervals from the study start date (VE_p) using the "unvaccinated" reference group. Points: mean from 100 simulations; error bars: 95%CI from 100 simulations. (C) as (A) but using the "recently vaccinated" reference group. (D) as (B) but using the "recently vaccinated" reference group. VE_t^*: simulated vaccine-induced protection at two-week time intervals since vaccine administration. VE_p^*: period vaccine effectiveness estimated under the assumption of reporting ratio equal to 100%.

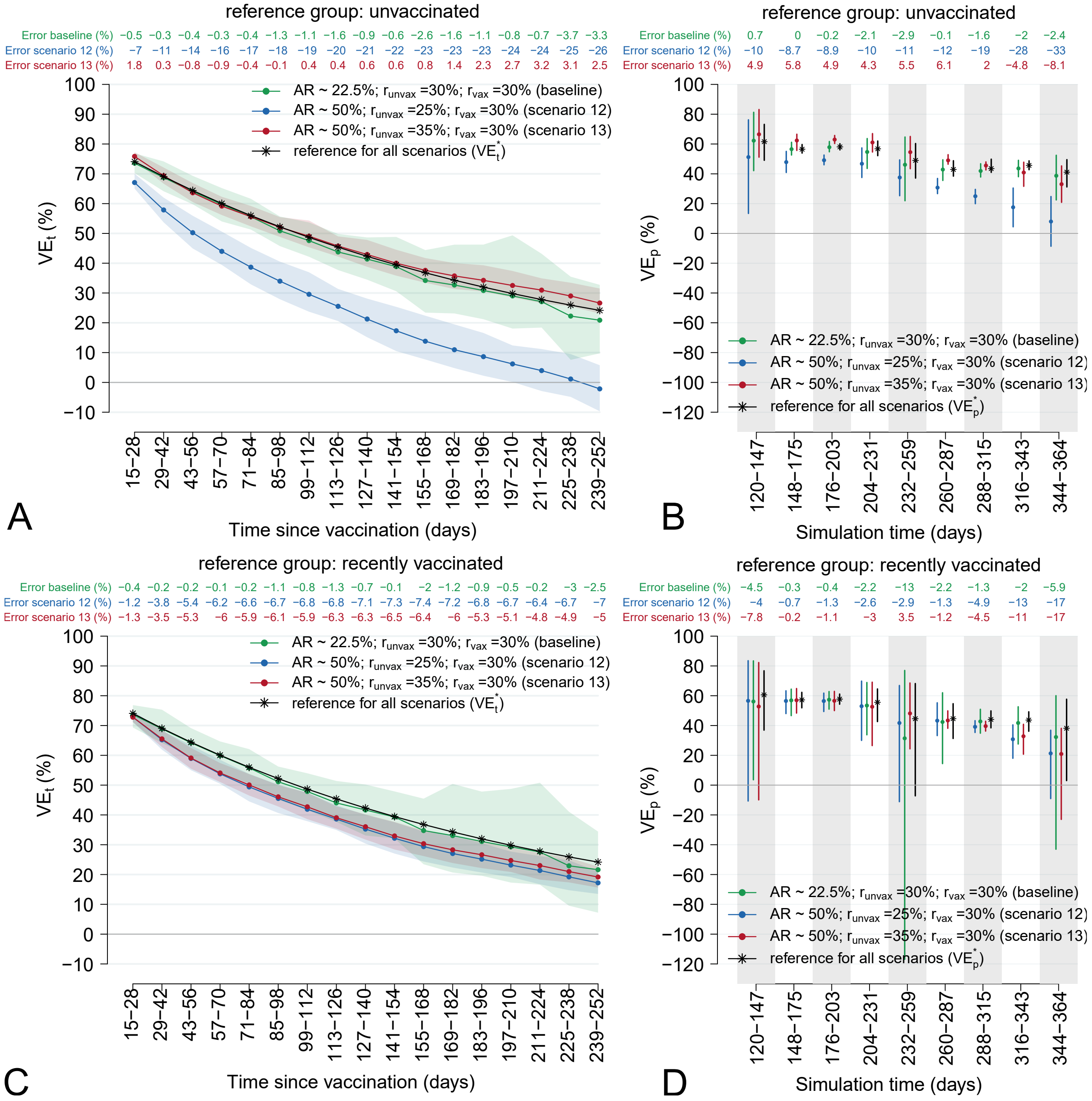

Figure S8. Changes in vaccine effectiveness (VE) estimates against notified infection considering heterogeneous reporting among unvaccinated and vaccinated and higher attack rate (50%) compared to the baseline (scenarios 12 and 13). (A) Estimates of VE at different time intervals from the vaccine administration (VE_t) using the "unvaccinated" reference group. Solid lines: mean from 100 simulations; shaded areas: 95%CI from 100 simulations. (B) Estimates of VE at different time intervals from the study start date (VE_p) using the "unvaccinated" reference group. Points: mean from 100 simulations; error bars: 95%CI from 100 simulations. (C) as (A) but using the "recently vaccinated" reference group. (D) as (B) but using the "recently vaccinated" reference group. VE_t^*: simulated vaccine-induced protection at two-week time intervals since vaccine administration. VE_p^*: period vaccine effectiveness estimated under the assumption of reporting ratio equal to 100%.

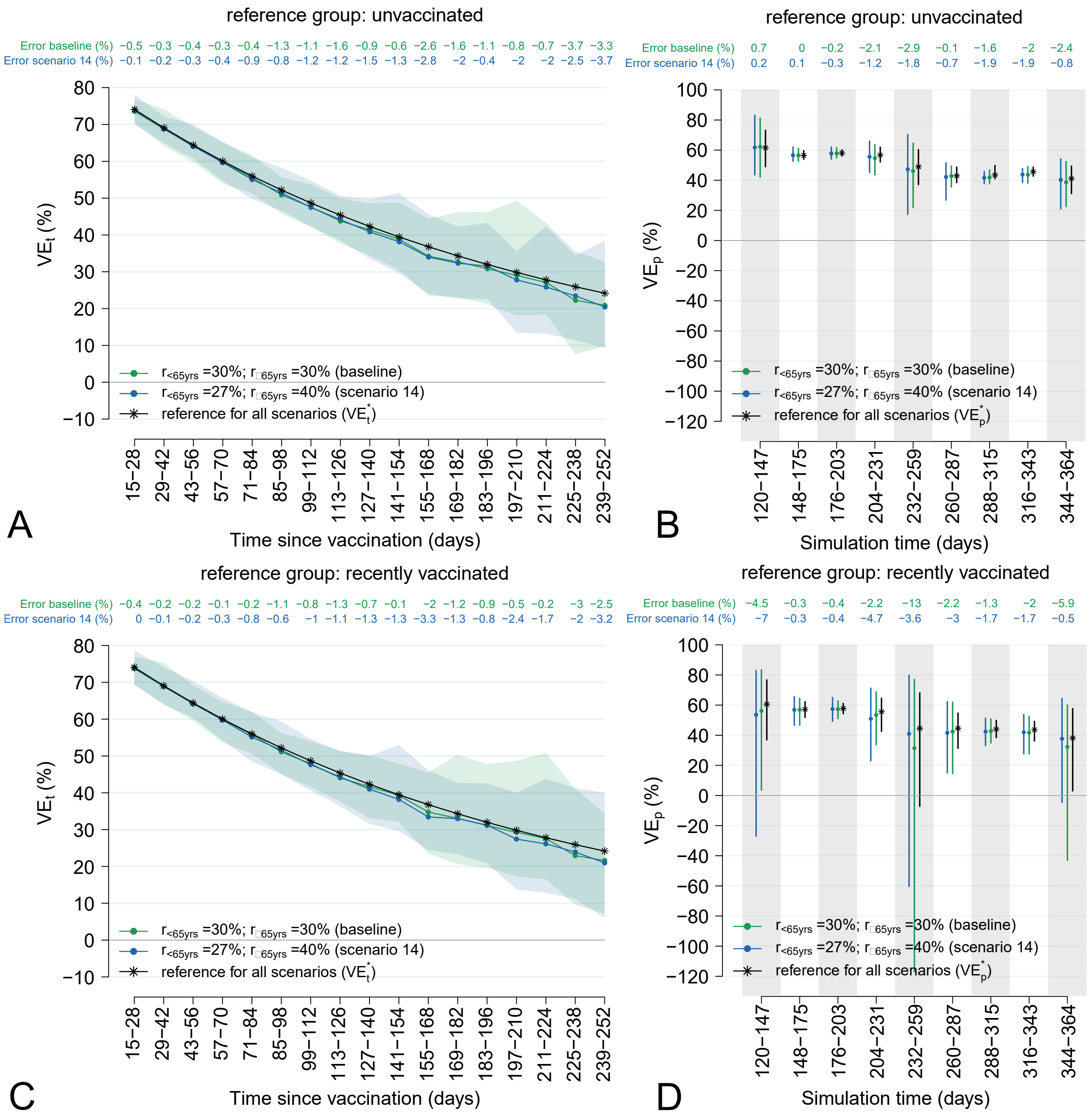

Figure S9. Changes in vaccine effectiveness (VE) estimates against notified infection considering heterogeneous reporting ratio (scenarios 14). (A) Estimates of VE at different time intervals from the vaccine administration (VE_t) using the "unvaccinated" reference group. Solid lines: mean from 100 simulations; shaded areas: 95%CI from 100 simulations. (B) Estimates of VE at different time intervals from the study start date (VE_p) using the "unvaccinated" reference group. Points: mean from 100 simulations; error bars: 95%CI from 100 simulations. (C) as (A) but using the "recently vaccinated" reference group. (D) as (B) but using the "recently vaccinated" reference group. VE_t^*: simulated vaccine-induced protection at two-week time intervals since vaccine administration. VE_p^*: period vaccine effectiveness estimated under the assumption of reporting ratio equal to 100%.
